# PAX8 expression in high-grade serous ovarian cancer positively regulates attachment to ECM via Integrin β3

**DOI:** 10.1186/s12935-019-1022-8

**Published:** 2019-11-20

**Authors:** Amata Amy Soriano, Tiziana de Cristofaro, Tina Di Palma, Serena Dotolo, Priyanka Gokulnath, Antonella Izzo, Gaetano Calì, Angelo Facchiano, Mariastella Zannini

**Affiliations:** 1 IEOS, Institute of Experimental Endocrinology and Oncology ‘G, Salvatore’-National Research Council, Naples, Italy; 2 Dpt. of Molecular Medicine and Medical Biotechnology, University of Naples Federico II, Naples, Italy; 3 Present Address: IRCCS Casa Sollievo della Sofferenza, Cancer Stem Cells Unit, ISReMIT, San Giovanni Rotondo, Foggia, Italy; 4 ISA, Institute of Food Science-National Research Council, Avellino, Italy

**Keywords:** PAX8, Ovarian cancer, Fallopian tube epithelial secretory cells, Integrins, Integrin β3

## Abstract

**Background:**

Ovarian cancer is the third most common cause of death among gynecologic malignancies worldwide. Understanding the biology and molecular pathogenesis of ovarian epithelial tumors is key to developing improved prognostic indicators and effective therapies. We aimed to determine the effects of PAX8 expression on the migrative, adhesive and survival capabilities of high-grade serous carcinoma cells.

**Methods:**

PAX8 depleted Fallopian tube secretory cells and ovarian cancer cells were generated using short interfering siRNA. *Anoikis* resistance, cell migration and adhesion properties of PAX8 silenced cells were analyzed by means of specific assays. Chromatin immunoprecipitation (ChIP) was carried out using a PAX8 polyclonal antibody to demonstrate that PAX8 is able to bind to the 5′-flanking region of the ITGB3 gene positively regulating its expression.

**Results:**

Here, we report that RNAi silencing of PAX8 sensitizes non-adherent cancer cells to *anoikis* and affects their tumorigenic properties. We show that PAX8 plays a critical role in migration and adhesion of both Fallopian tube secretory epithelial cells and ovarian cancer cells. Inhibition of PAX8 gene expression reduces the ability of ovarian cancer cells to migrate and adhere to the ECM and specifically to fibronectin and/or collagen substrates. Moreover, loss of PAX8 strongly reduces ITGB3 expression and consequently the correct expression of the αvβ3 heterodimer on the plasma membrane.

**Conclusions:**

Our results demonstrate that PAX8 modulates the interaction of tumor cells with the extracellular matrix (ECM). Notably, we also highlight a novel pathway downstream this transcription factor. Overall, PAX8 could be a potential therapeutic target for high-grade serous carcinoma.

## Background

Ovarian cancer is known for being the most lethal gynecological malignancy [1] and includes a heterogenous group of tumors involving the ovary. High-Grade Serous Ovarian Cancer (HGSC), the most incident and fatal subtype of ovarian cancer, contributes to ~ 70% of all ovarian cancer deaths [1]. The high mortality is attributed to the late diagnosis, more often after the peritoneal spread of HGSC [2]. It is established as one of the most malignant ovarian cancer diagnosed with only 30% patients demonstrating 5-year survival in spite of recent advances in chemotherapy [2]. Therefore, it is critical to understand the tumorigenic and metastatic processes that lead to the development of HGSC. Though the site of origin of this cancer could well be both ovary and Fallopian tubes, due to the mounting evidence [3–7] the Fallopian tube epithelium is now popularly accepted to be the primary tumorigenic site for the majority of HGSC cases. Overwhelming support to this theory is provided by the presence of Serous Tubal Intraepithelial Carcinoma (STIC) in the Fallopian tube in ~ 68% of HGSC patients [8]. Another important evidence that further strengthen this theory is the presence of the Fallopian Tube Epithelium (FTE) lineage specific marker PAX8 in almost all HGSC [9–12]. PAX8 is a member of the evolutionarily well-conserved paired-box gene family composed of nine transcription factors known for their role in embryogenesis with their protein expression tightly controlled temporally and spatially. PAX8 has been demonstrated to be crucial in determining cell fate during the development of thyroid, kidney, brain, eyes and Mullerian system; in adult normal tissues, it is expressed in kidney, thyroid and Fallopian tubes and not in the ovarian epithelium [13–15]. This could be particularly relevant in the development of HGSC because PAX8 is neither lost, mutated or overexpressed but is functionally retained possibly due to a selective advantage that it confers upon HGSC tumor cells. Indeed, PAX8 belongs to a class of lineage-survival genes that are required for both normal development of specific tissues and for cancer cell proliferation/survival. Therefore, understanding PAX8 downstream network in HGSC could give important cues in unravelling new therapeutic targets. In recent years, the pro-tumorigenic role of PAX8 in ovarian cancer has been well demonstrated [16, 17] and PAX8 signaling network is under investigation to better tackle HGSC [18–20]. As suggested by recent studies, it is possible that new targets become available for PAX8 in ovarian cancer due to the reprogramming of PAX8 cistrome by epigenetic modifications that occur during tumorigenesis [19, 21, 22]. Actually, we believe that PAX8 possibly continues to exert its transcriptional activity on its physiological targets and may also function on newly available targets after the tumorigenic hits.

Ovarian cancers, unlike other cancers, metastasize mostly without vasculature but as spheroidal clusters or single cells and get seeded in neighboring peritoneum or bladder [23]. A possible reason for this aggressive dissemination could be because the first epithelial mesenchymal transition (EMT) occurs in STIC of FTE that spread to the ovary as HGSC. It is interesting to note that cell-adhesion was one of the important processes regulated by PAX8 revealed in our previous study [18]. It is an established observation of how cell-adhesion is completely dysregulated in highly metastatic cancers. Since PAX8 is an important connecting link between the non-malignant FTE secretory cells, the STIC (now reported as malignant [24]) and malignant HGSC, we believe it is crucial to revisit PAX8 signaling pathways in these contexts.

Here, we report that PAX8 is involved in migration and adhesion of both Fallopian tube secretory epithelial cells and ovarian cancer cells; in addition, it confers resistance to *anoikis* or detachment-induced apoptosis leading to EMT. Interestingly, inhibition of PAX8 gene expression in ovarian cancer cells decreases tumor cell adhesion to fibronectin and collagen.

Furthermore, loss of PAX8 strongly reduces ITGB3 expression and consequently the correct expression of the Integrin αvβ3 heterodimer on the plasma membrane. Integrin β3 has been already implicated in a wide variety of functions, including platelet aggregation and thrombosis, implantation, placentation, angiogenesis, bone remodeling, and tumor progression [25]. Amongst integrins that have been identified as important mediators of ovarian cancer metastasis, the heterodimer Integrin αvβ3 holds a significant position [26, 27].

This is the first study reporting the correlation between PAX8 and Integrins uncovering a novel functional pathway downstream of this transcription. Moreover, we suggest a possible role for PAX8 in the peritoneal dissemination of ovarian cancer cells by modulating cancer cells’ *anoikis*-susceptibility and the interaction of tumor cells with the extracellular matrix (ECM).

## Methods

### Cell lines and culture conditions

Human ovarian cancer cell line SKOV3 was provided by the CEINGE Cell Culture Facility (Naples, Italy). High-grade serous ovarian cancer cell lines KURAMOCHI (JCRB No. JCRB0098) and OVSAHO (JCRB No. JCRB1046) were obtained from the Japanese Collection of Research Bioresources Cell Bank (JCRB). These cell lines were maintained in RPMI-1640 medium supplemented with 10% fetal bovine serum and 1% penicillin/streptomycin (Euroclone, Italy). The human ovarian adenocarcinoma cell line PEA1 was purchased from Sigma-Aldrich and was grown in RPMI-1640 medium supplemented with 10% fetal bovine serum, 2 mM glutamine, 2 mM sodium pyruvate and 1% penicillin/streptomycin (Euroclone, Italy).

### Tissue samples and primary human Fallopian tube secretory epithelium ex vivo culture system

Primary human Fallopian tube secretory cells (Primary hFTSECs) were isolated from fresh Fallopian tube (FT) fimbria specimens obtained from the Department of Gynaecology of the AOU Federico II (Naples, Italy) with approval of the institutional review board. The human tissues used in this study were collected from surgical procedures for benign gynecological indications. Specifically, cases of inflammatory disease, infection, and extensive adhesions were excluded. The FT tissue was washed several times using 0.9% NaCl solution and 1% penicillin/streptomycin (Euroclone, Italy) until all traces of blood was completely removed. The tissue was further washed in CHANG MEDIUM C (IrvineScientific, USA), to help with its stabilization, and it was dissected into very small 1 mm sized pieces with sterilized scalpel and needle. The tissue was incubated at 37 °C overnight for enzymatic digestion using 0.8 mg/ml of collagenase I (Sigma, Germany) on 60 mm culture dishes supplemented with DMEM-F12 (Euroclone, Italy) with reduced serum 5% fetal bovine serum (Euroclone, Italy) and 1% penicillin/streptomycin (Euroclone, Italy), to dissociate into single cells. Supernatant collected after O/N digestion was centrifuged at 1200 rpm for 5 min and the pellet composed of dissociated cells was plated on collagen coated 60 mm plate supplemented with DMEM-F12 (Euroclone, Italy), 2% Ultroser G serum (PALL, France) and 1% penicillin/streptomycin (Euroclone, Italy). After a visual estimation of cells that grew in plates, epithelial-like cells that grew in clusters were carefully trypsinized using clonal cylinders and re-plated onto a fresh 60 mm collagen coated culture plate. These cells, named Primary hFTSECs, were analyzed for Fallopian tube secretory epithelial cell markers such as PAX8 (kindly provided by R. Di Lauro) and OVGP1 (Oviductin sc-46432, Santa Cruz, USA) using immunofluorescence. At every passage, the cells were verified for these markers and only then used for experiments.

### Cell culture transfection

In all the experiments, PAX8 expression was transiently downregulated by means of RNA interference. For migration, adhesion and immunofluorescence assays Primary hFTSEC, SKOV3, KURAMOCHI, OVSAHO and PEA1 cell lines were transfected with 5 nM PAX8 siRNA (Ambion, Life Techonologies, siRNA ID s15403) or siRNA Non-Targeting (Ambion, Life Technologies, siRNA ID 4390843) as scramble control (siCTR) for 48 h using the Lipofectamine RNAiMAX transfection reagent (Invitrogen, USA) according to the manufacturer’s protocol. Other PAX8 siRNAs used to confirm the specificity of the effects were siRNA ID s15404 and siRNA ID s15405 (Additional file 3: Fig. S2) both from Ambion, Life Technologies.

For the ITGB3 rescue experiment, plasmid pcDNA3.1-beta-3 was a gift from Timothy Springer (AddGene plasmid # 27289) [28] and it was transiently transfected in KURAMOCHI and PEA1 cells as described above.

### RNA extraction, cDNA preparation and Real Time qPCR

Total RNA was extracted using the RNeasy Mini kit (Qiagen, Germany). The cDNA was synthesized using the iScript cDNA Synthesis kit (BIORAD, Hercules, CA). Real-time qPCR analysis was performed using the IQ™ SYBR Green PCR Master Mix (BIORAD, Hercules, CA) in a CFX96 Real-Time PCR Detection System (BIORAD, Hercules, CA) for the following genes using gene-specific primers. Sequences of primers used in qRT-PCR:

PAX8 5′-CCCTTCCAACACGCCACT-3′ (fwd); 5′-CTGCTTTATGGCGAAGGGTG-3′ (rev)

ITGB3 5′-CTCATATAGCATTGGACGGAAGG-3′ (fwd); 5′-ACATTTTCAGTCACTGCAAAGAT-3′ (rev)

ITGAV 5′-CGGATGTTTCTTCTCGTGGG-3′ (fwd); 5′-CCTCACAGATGCTCCAAACC-3′ (rev)

ABL 5′-TGGAGATAACACTCTAAGCATAACTA-3′ (fwd); 5′-GATGTAGTTGCTTGGGACCCA-3′ (rev)

VIM. 5′-GAATACCGGAGACAGGTGCAG-3′ (fwd); 5′-CGGCCAATAGTGTCCTGGTAG-3′ (rev)

FIBR. 5′-CTACCAAGGCTGGATGATGGTGG-3′ (fwd); 5′-GGAGCAGGTTCCCTCTGTTG-3′ (rev)

BCL2 5′-GCCCTGTGGATGACTGAGTA-3′ (fwd); 5′-AGGGCCAAACTGAGCAGAG-3′ (rev)

ZEB2 5′-CCAGAAGCCACGATCCAGAC-3′ (fwd); 5′-ACTGCATGACCATCGCGTTCC-3′ (rev).

For each gene, values are mean ± SD of three independent experiments, normalized by the expression of an housekeeping gene (ABL) and expressed as a percentage of the value measured in control cells. To calculate the relative expression levels we used the 2-DDCT methods [29].

### Protein extracts and immunoblotting

Cells were washed twice with ice-cold phosphate-buffered saline (PBS) and lysed in JS buffer containing 50 mM Hepes pH 7.5, 150 mM NaCl, 5 mM EGTA pH 7.8, 10% glycerol, 1% Triton, 1.5 mM MgCl2, 1 mM dithiothreitol (DTT), 1 mM phenylmethylsulfonyl fluoride (PMSF). The protein concentration was determined using the Bio-Rad protein assay (Bio-Rad Laboratories, Inc., Hercules, CA). For Western blot analysis, proteins were separated on SDS–PAGE, gels were blotted onto Immobilon P (Millipore, Bredford, MA, USA) for 2 h and the membranes were blocked in 5% nonfat dry milk in Tris–buffered saline for 2 h or overnight before the addition of the antibody for 1 h. The primary antibodies used were: anti-PAX8 (kindly provided by R. Di Lauro), anti-Vinculin (Santa Cruz Biotech., sc-7649), anti GAPDH (Santa Cruz Biotech., sc-32233), anti-Bcl-2 (CST, 2870), anti-Bax (CST, 2772). The filters were washed three times in Tris–buffered saline plus 0.05% Tween 20 before the addition of horseradish peroxidase-conjugated secondary antibodies for 45 min. Horseradish peroxidase was detected with ECL (Pierce, Thermo Scientific).

### Migration assay

Migration assay was performed using Ibidicell migration technology (Ibidi, Martinsried, Germany). PAX8 was silenced in Primary hFTSECs, SKOV3, KURAMOCHI, OVSAHO and PEA1 cell lines as described before. After 24 h, both scramble and PAX8 silenced cells were seeded in each chamber at a density of 3 × 10^5^ cells/reservoir in 70 μl of normal medium for 24 h. The medium was then replaced with fresh medium and cells were treated with 10 ug/ml of Mitomycin C (Sigma M4287-2MG) for 1 h at 37 °C. After the incubation, the chambers were removed and cells were further incubated in normal medium. Cells were photographed (1:1 magnification) and the area covered by the cells within a defined area in the gap was measured using the NIH ImageJ (http://rsb.info.nih.gov/ij) software.

For the ITGB3 rescue experiment, PAX8 was silenced in KURAMOCHI and PEA1 cells as described before and transiently transfected with plasmid pcDNA3.1-beta-3 encoding for the Integrin β3 protein.

### Adhesion assay

Coverslips were coated with 10 µg/ml of Fibronectin (EDM Millipore Corp., USA) or Collagen I (EDM Millipore Corp., USA) in PBS 1× for 1 h at 37 °C. PAX8 was silenced in Primary hFTSECs, SKOV3, KURAMOCHI, OVSAHO and PEA1 cell lines as described before. After 48 h, 40 × 10^3^ of both scramble and PAX8 silenced cells were plated on the top of coated coverslips in triplicates for 2 h at 37 °C. After incubation, coverslips were washed with PBS 1×, fixed in 4% paraformaldehyde for 10 min and nucleus stained with HOECHST. The experiment was repeated three times (n = 3) for each cell line. Images were acquired using Confocal microscope (ZEISS LSM 700). For each coverslip, 10 images were acquired and analyzed using the ImageJ software.

For the ITGB3 rescue experiment, PAX8 was silenced in KURAMOCHI and PEA1 cells as described before and transiently transfected with plasmid pcDNA3.1-beta-3 encoding for the Integrin β3 protein.

### *Anoikis* assay

To assess the *anoikis* activity, 1 × 10^4^ of both scramble and PAX8 silenced Kuramochi cells 24 h after transfection were plated in triplicate on ultra-low attachment 96-well plates under regular culture conditions and on adherent 96-well plates, as control. Cell viability was detected 24 h and 48 h later using the MTS reagent (Promega, G3580). The viability ratio of cells grown in the two different wells was calculated using OD_anoikis well_/OD_control well_.

### Immunofluorescence and Confocal Laser Scanning Microscopy

After 24 h of transfection with siCTR and siPAX8 as described before, 50 × 10^3^ of Primary hFTSECs and KURAMOCHI cells were plated on glass coverslips and maintained in culture for 24 h at 37 °C. Cells were fixed in 4% paraformaldehyde in PBS 1× for 20 min at RT and incubated for 30 min in 10% FBS in PBS 1×. Coverslips were subsequently incubated for 1 h with mouse monoclonal anti-αvβ3 LM609 (Millipore Corp, USA) and rabbit polyclonal anti-PAX8 diluted to 1:100 and 1:1000 in 4% FBS in PBS 1×, respectively. After PBS washes, cells were incubated for 30 min with Alexa Fluor-546 goat anti-mouse IgG (Vinci Biochem) and Alexa Fluor-488 goat anti-rabbit IgG (Vinci Biochem) both diluted to 1:200 in 4% FBS in PBS 1×. After the final washes with PBS 1×, coverslips were mounted on microscope slides using a 50% solution of glycerol in PBS 1× with Hoechst (1:3000). Experiments were carried out on an inverted and motorized microscope (Axio Observer Z.1) equipped with a 63×/1.4 Plan-Apochromat objective. The attached laser-scanning unit (LSM 700 4× pigtailed laser 405-488-555-639; Zeiss, Jena, Germany) enabled confocal imaging. For excitation, 405, 488 and 555 nm lasers were used. Fluorescence emission was revealed by Main Dichroic Beam Splitter and Variable Secondary Dichroic Beam Splitter. Double and/or triple staining fluorescence images were acquired separately using ZEN 2012 software in the blue, green and/or red channels at a resolution of 1024 × 1024 pixels, with the confocal pinhole set to one Airy unit and then saved in TIFF format.

### Chromatin immunoprecipitation assay

ChIP was performed as previously described [30]. Precleared chromatin from KURAMOCHI cells was incubated with 3 μg of affinity-purified rabbit polyclonal anti-PAX8 antibody (Thermo Scientific, PA1-112) or polyclonal anti-K-cadherin antibody as unrelated (Santa Cruz Biotechnology, sc-1503) and rotated at 4 °C for 16 h. Thereafter, the immunoprecipitated DNAs were amplified by quantitative real-time PCR with the following primers:

ITGB3 5′-CAGCCTTAAGGTCTTTGTGTTG-3′ (fwd); 5′-TCAGACCATGATGTGAAGCAG-3′ (rev).

### Network analysis by bioinformatics tools

Network analysis has been performed using Cytoscape v3.6 Core [http://www.cytoscape.org, Christian T. Bioinformatics. 2010] one of the most popular open-source software for visualizing, analyzing and modelling biological networks, both for protein and gene networks [31–33]. The latest generation of Cytoscape (version 3.0 and later) makes it possible to integrate the genomic information (RNA-seq data) with the biological networks applying some specific tools based on study of genomic data [34]. Data of 302 RNA-seq genes up/down-regulated before and after PAX8 silencing in HGSC were imported on Cytoscape in excel format, pre-processed using “NetworkAnalyzer” and “ClusterOne”, both implemented as default Cytoscape plugins to understand the organization and directionality of network [35, 36]. PAX8 has been used as the starting point to obtain the biological network. The distribution of up/down-regulated genes and network organization have obtained taking into consideration the following parameters: gene name, FPKM1 (value after PAX8 silencing), FPKM2 (value before PAX8 silencing), log2 and abs(log2) with cutoff < 2, to delete the self-loops and miRNAs data. The analysis of these data was performed applying “GenomeSpace”, “Signor 2.0″ and “MCODE” plugins, to realize and visualize the molecular network at graphical level exploring the changes that occur before and after PAX8 silencing [37–39]. Image of molecular networks were saved in*.png* format on Cytoscape platform using 600 DPI and 500% of resolution to generate publication-quality images from network views. The thickness of the arrow indicates the strength of the interaction and the color of the arrows indicate the biological importance of the interactions. The detailed legend for Cytoscape analysis is reported as Additional file 1: Table S1.

## Results

### PAX8 inhibition affects migration and adhesion in Fallopian tube secretory cells and ovarian cancer cells

**Fig. 1:**
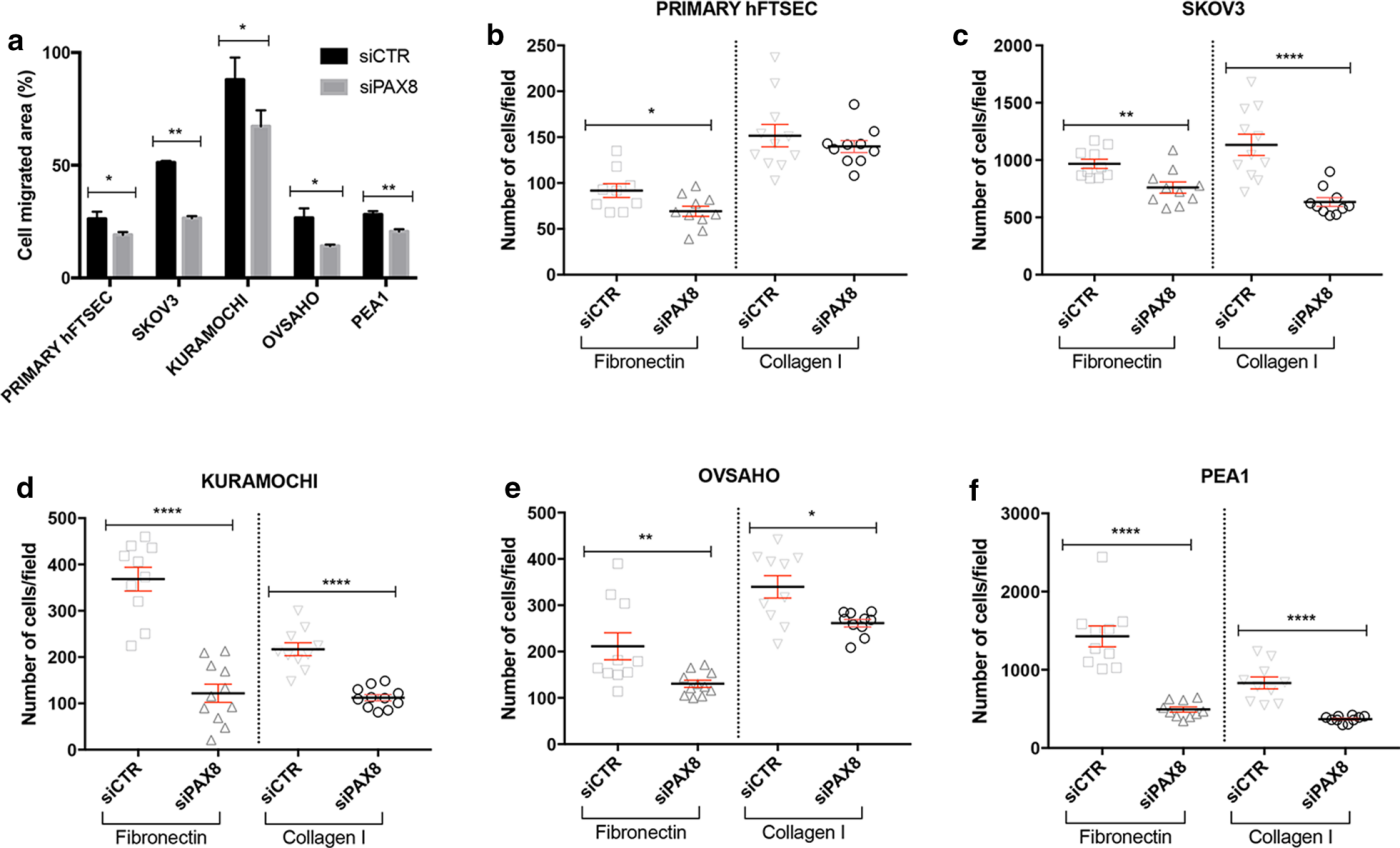
PAX8 silencing impairs migration and adhesion of Primary hFTSEC, SKOV3, KURAMOCHI, OVSAHO and PEA1 cells. **a** The cell migrated area was measured 48 h after transient transfection with PAX8 siRNA (grey bars) or scramble siRNA (black bars). **b**–**f** The adhesion ability to Fibronectin or Collagen I of Primary hFTSEC, SKOV3, KURAMOCHI, OVSAHO and PEA1 cells was analyzed 48 h after transient transfection with PAX8 siRNA or scramble siRNA. In all the experiments, the values are mean ± SD of three independent experiments normalized with respect to the cells transfected with the scramble siRNA. *p* value was calculated by *t*-test *p *≤ 0.1

Cell migration requires dynamic interactions between cells and their substratum on which they attach and move. Changes in the adhesion molecule repertoire may correspond to changes in migratory properties conferring a more invasive phenotype to cancer cells. To explore the migratory ability of Fallopian tube secretory cells and epithelial ovarian cancer cells, migration assays were performed using the Ibidi cell migration technology after RNAi of PAX8 (see “Methods” and Additional file 2: Fig. S1). Our results clearly highlight the effect of PAX8 loss on the migratory properties of the cells demonstrated by the significant reduction in the cell migrated area of PAX8-silenced cells with respect to control cells both in Primary hFTSEC and all epithelial ovarian cancer cells that were analyzed (SKOV3, KURAMOCHI, OVSAHO and PEA1) (Fig. 1a).

In parallel, to understand the consequences of PAX8 loss on cell–matrix adhesion the ability of the cells to adhere to different extracellular matrices (ECM) was addressed. RNAi of PAX8 was performed as described (see “Methods”) and the adhesive abilities of the cells were evaluated through cell adhesion assays using Fibronectin and Collagen I as substrates to mimic the ECM. Upon PAX8 silencing, all cell types exhibited a significantly impaired adhesion ability when cultured on Fibronectin and Collagen I-coated coverslips (Fig. 1b–f).

RNAi of PAX8 performed with two additional siRNA (see “Methods”) confirmed the impaiment of migration and adhesion (Additional file 3: Fig. S2).

### The presence of PAX8 correlates with the achievement of *anoikis* resistance of ovarian cancer cells

**Fig. 2:**
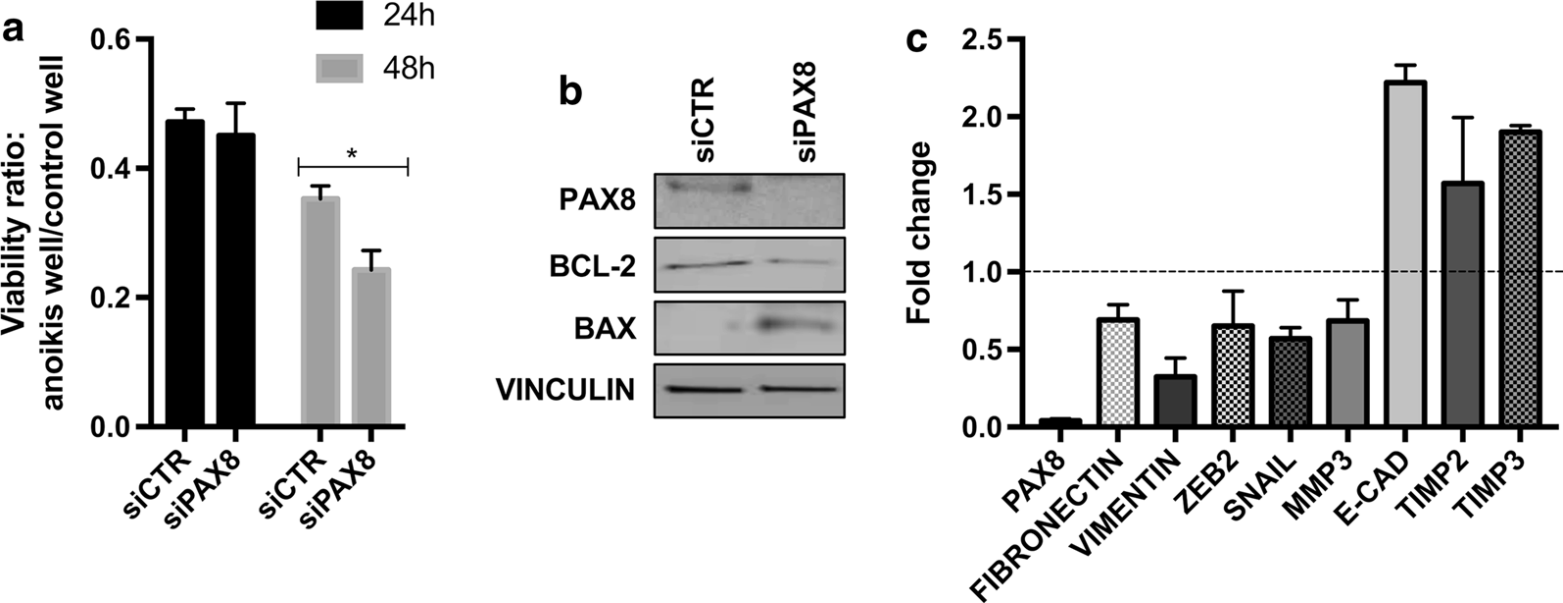
RNAi of PAX8 brings to an increased sensitivity to *anoikis* of ovarian cancer cells and reduced expression of EMT markers. **a** Following RNAi of PAX8, the survival percentage of anchorage-independent KURAMOCHI cells was compared with that of cells under adherent condition at 24 h and 48 h. Values are mean ± SD of three independent experiments normalized with respect to the cells transfected with the scramble siRNA. *p*-value was calculated by *t*-test *p *≤ 0.1. **b** Western blots to evaluate the protein levels of Bcl-2 and Bax after PAX8 depletion. **c** The expression levels of a panel of mesenchymal markers were measured by qRT-PCR in KURAMOCHI cells 48 h after RNAi of PAX8. The values are mean ± SD of three independent experiments in duplicate, normalized by the expression of ABL and expressed as fold change with respect to the siCTR cells, whose value was set at 1.0. *p*-value was calculated by *t*-test 0.001 ≤ *p *≤ 0.1

Epithelial cells are protected by *anoikis* when they are adherent to ECM proteins. It is well known that cancer cells develop *anoikis* resistance due to several mechanisms that at the end help metastatic cancer cells to invade distant organs. Since we have demonstrated that PAX8 is involved in cell migration and adhesion, we asked whether it could also affect *anoikis* resistance of ovarian cancer cells. To this end, KURAMOCHI cells were transfected with siCTR and siPAX8 as described (see “Methods”), cultured under anchorage-independent conditions for 24 h and 48 h and viability of the cells was assessed by MTT assay and compared with the cells cultured simultaneously under adherent conditions. As shown in Fig. 2a, PAX8 silenced cells show a lower percentage of cell-survival compared to that of the parental cells indicating that the loss of PAX8 brings to an increased sensitivity to *anoikis* of ovarian cancer cells and accordingly decreased bcl-2 and increased Bax expression (Fig. 2b). Moreover, the probability of cell transition to a less mesenchymal phenotype was investigated and known mesenchymal markers such as vimentin, fibronectin, ZEB2 and others were analyzed (Fig. 2c).

### PAX8 positively regulates Integrin β3 gene expression

**Fig. 3:**
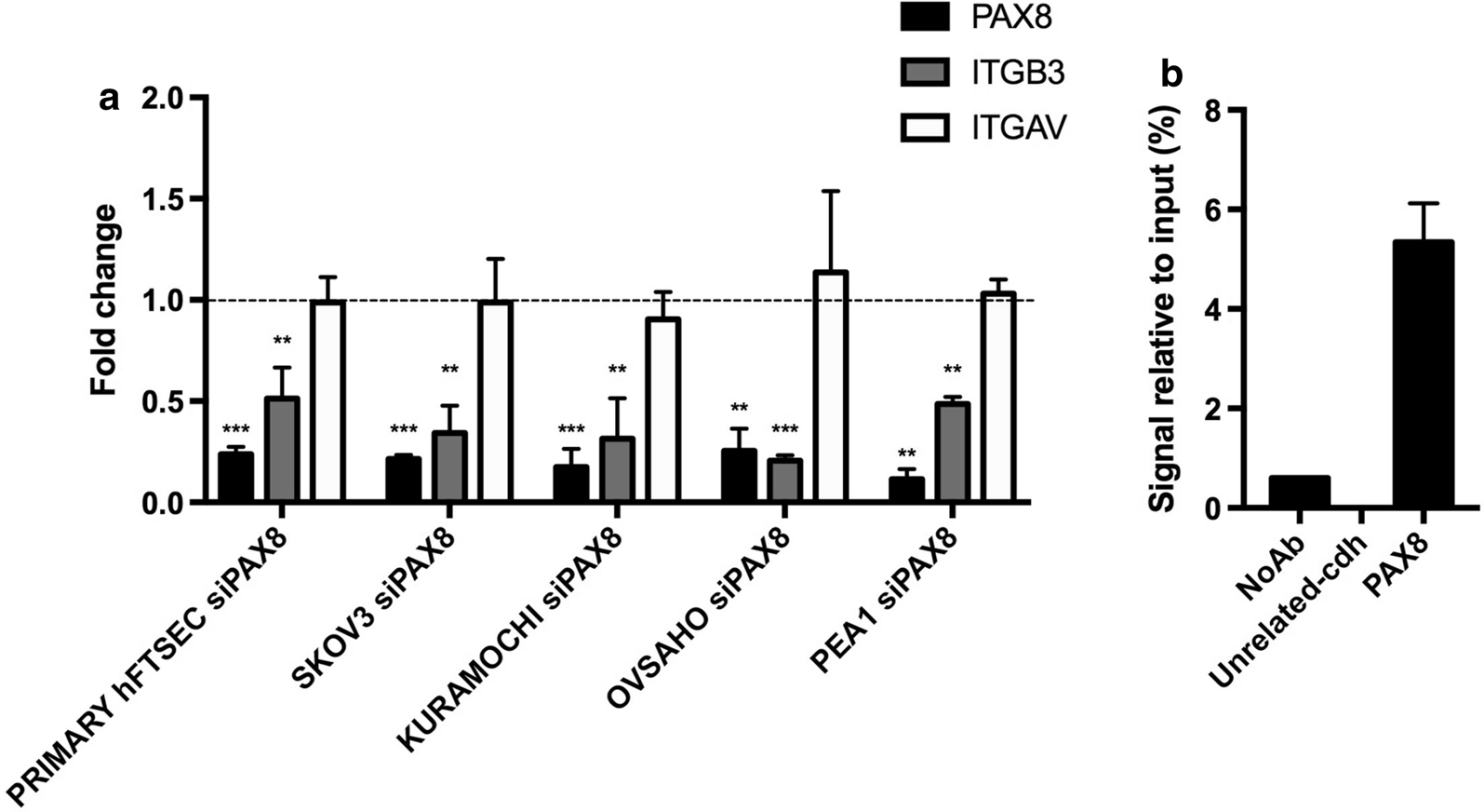
PAX8 regulates the expression of the ITGB3 gene. **a** PAX8, ITGB3 and ITGAV expression levels were measured by qRT-PCR in Primary hFTSEC, SKOV3, KURAMOCHI, OVSAHO and PEA1 48 h after RNAi of PAX8. The values are mean ± SD of three independent experiments in duplicate, normalized by the expression of ABL and expressed as fold change with respect to the siCTR cells, whose value was set at 1.0. *p*-value was calculated by *t*-test 0.001 ≤ *p *≤ 0.1. **b** Chromatin immunoprecipitation assay was performed to determine the binding of PAX8 to the 5′-flanking regions of ITGB3. Chromatin was subjected to qPCR analysis using appropriate primers (see “Methods”)

Many experimental evidences demonstrated that deregulation of integrins or changes in their expression profile can contribute to cancer cells acquisition of tumorigenic properties. In our previous study [18], we reported that upon PAX8 silencing Integrin β3 (ITGB3) is amongst the genes significantly downregulated. Hence, we asked whether following PAX8 inhibition ITGB3 expression was modified also in normal Fallopian tube and in general in ovarian cancer cells. To this end, Primary hFTSEC, SKOV3, KURAMOCHI, OVSAHO and PEA1 cells were transfected with siCTR and siPAX8 as already described. Real-time qPCR analysis subsequently performed showed a good correspondence between PAX8 and Integrin β3 expression being the latter significantly decreased in all PAX8 silenced samples (Fig. 3a).

To get more insights on PAX8 regulation of ITGB3 gene expression, we asked whether the mechanism was direct. Initially, to determine whether PAX8 could directly bind to the regulatory genomic sequence of the ITGB3 gene we performed a computational analysis using the MatInspector Software (Genomatix). We searched for PAX8 binding sites in a region of about 2 Kb in the 5′-flanking region of the ITGB3 gene and we identified several PAX8 consensus sequences. To confirm the predictions of the MatInspector analysis, we carried out chromatin immunoprecipitation (ChIP) assays on KURAMOCHI cells using a PAX8 polyclonal antibody. Indeed, the ChIP result confirmed that PAX8 is able to bind to the regulatory region of ITGB3 in a position close to the start of transcription (Fig. 3b).

**Fig. 4:**
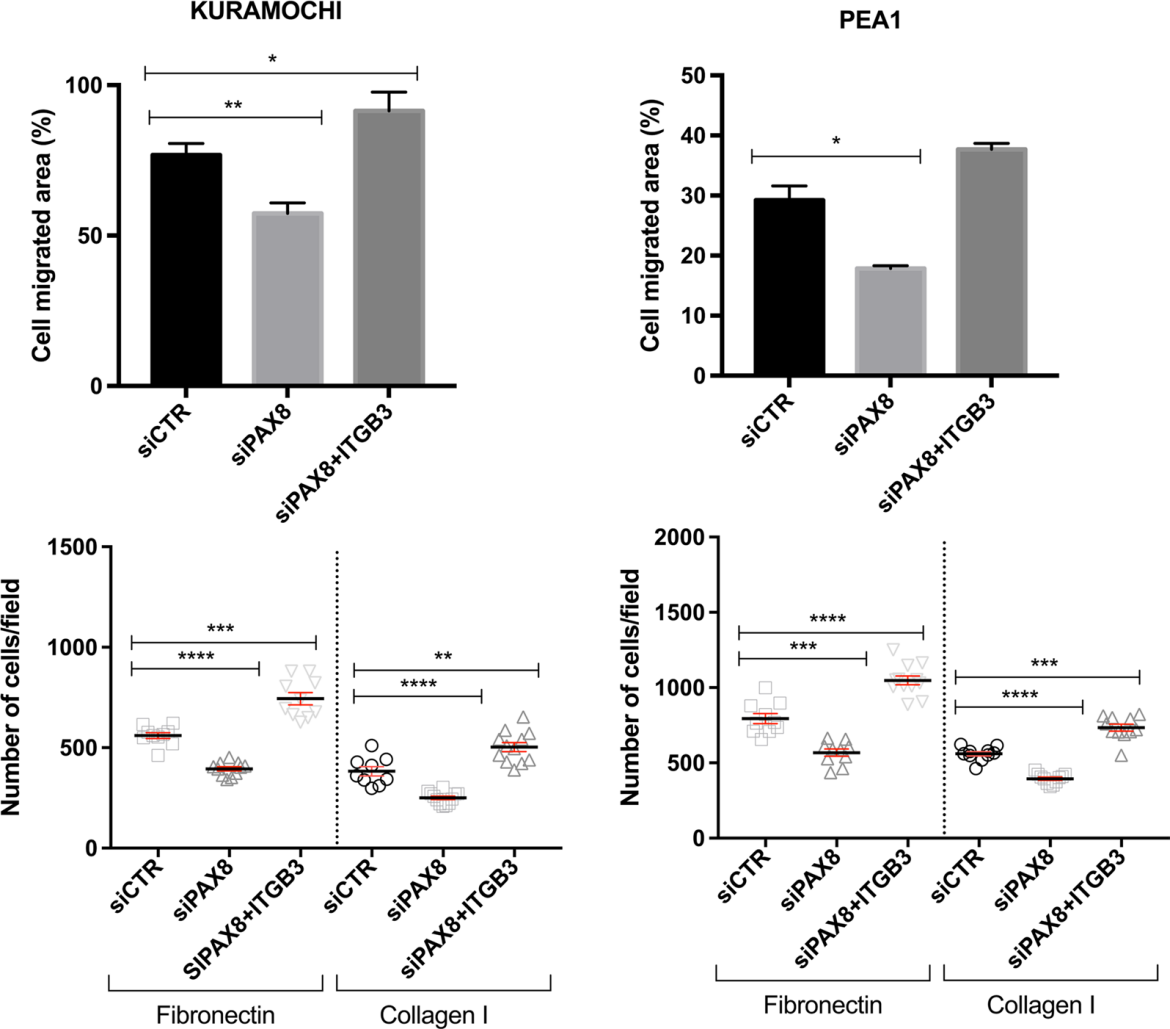
Integrin β3 overexpression is able to reverse the effects of PAX8 silencing. The cell migrated area and the adhesion ability to Fibronectin or Collagen I of KURAMOCHI and PEA1 cells was analyzed 48 h after transient transfection with scramble siCTR, siPAX8 or siPAX8 + ITGB3 expression vector. In all the experiments, the values are means ± SD of three independent experiments normalized with respect to the cells transfected with the scramble siRNA. *p*-value was calculated by *t*-test *p *≤ 0.1

Rescue experiments were finally conducted to confirm the role of Integrin β3 in migration and adhesion of ovarian carcinoma cells. Specifically, KURAMOCHI and PEA1 cells were transfected with siCTR, siPAX8 or siPAX8 + ITGB3 expression vector, respectively. Migration and adhesion assays revealed that the effects caused by PAX8 knockdown could be reversed by Integrin β3 overexpression (Fig. 4).

Taken together, our results show for the first time a correlation between PAX8 and the Integrins family of receptors suggesting a novel functional pathway downstream of this transcription.

### Loss of PAX8 affects plasma membrane expression of the Integrin αvβ3

Integrin β3 is reported to bind to only two other α integrin subunits: Integrin αIIb and Integrin αv. The presence of αIIbβ3 dimers is restricted to cells of the megakaryocyte lineage and is required for platelet aggregation [40] while αvβ3 dimers are present on proliferating endothelial cells and some cancer cells. There is evidence for the role of αvβ3 heterodimer in multiple mechanisms of tumor growth and invasion, including interaction with ECM components, matrix metalloproteinase 2, platelet-derived growth factor, insulin, VEGF receptors, and prevention of apoptosis [41]. Recently, αvβ3 heterodimer has been observed in ovarian cancer cells and its potential as a therapeutic target for blocking tumor-induced angiogenesis is being considered [27].

**Fig. 5:**
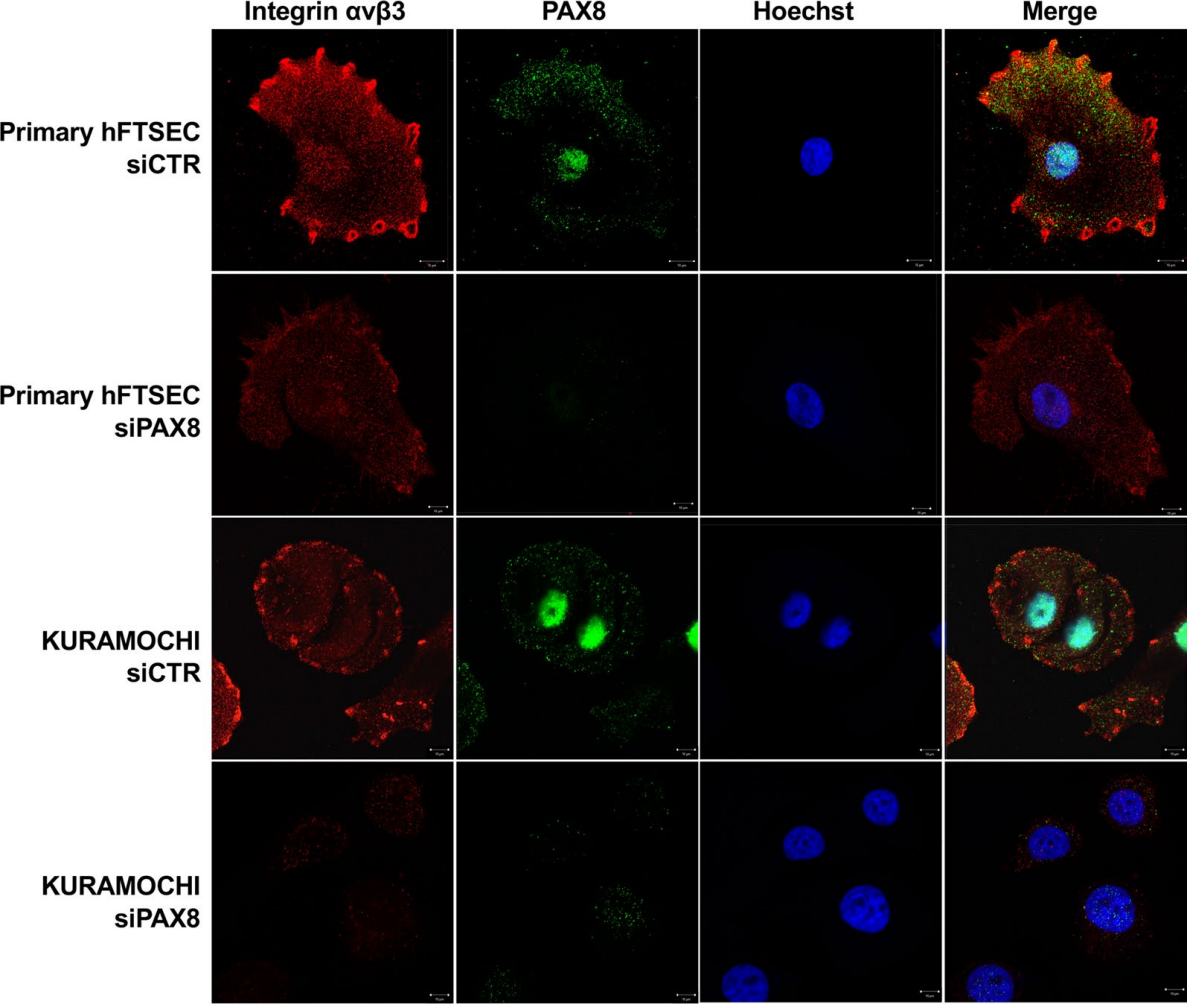
PAX8 knockdown reduces the expression of αvβ3 on the plasma membrane. Primary hFTSEC and KURAMOCHI cells were transfected with scramble siRNA (siCTR) and PAX8 siRNA (siPAX8) and after 24 h were plated on coverslips and maintained in culture for 24 h. The confocal fluorescence analysis was performed for Integrin αvβ3 (red channel) and PAX8 (green channel). Hoechst (blue channel) was used to locate the nuclei (scale bar 10 μm). The images are representative of three independent experiments

Since the functionality of integrins depends upon their expression on the plasma membrane we reasoned that the reduced expression of ITGB3 observed upon PAX8 silencing could result in a decrease expression of Integrin β3 and likely of the αvβ3 heterodimer on the cell surface. To test this hypothesis, we performed direct immunofluorescence on Primary hFTSECs and KURAMOCHI cells before and after PAX8 silencing. As showed in Fig. 5, the lower intensity of PAX8 signal in siPAX8-Primary hFTSECs and siPAX8-KURAMOCHI cells was a confirmation of PAX8 silencing. Interestingly, a significant downregulation of the Integrin αvβ3 signal on the cell membrane was observed in good correlation with PAX8 inhibition indicating an important contribution of PAX8 for the presence of the αvβ3 heterodimer on the surface of ovarian cancer cells.

### Cluster analysis of PAX8-Integrin β3 network

To analyze the PAX8-Integrin β3 network that could govern migration and adhesion in ovarian carcinoma, we re-analyzed our previous RNA-seq data using the Cytoscape platform.

**Fig. 6:**
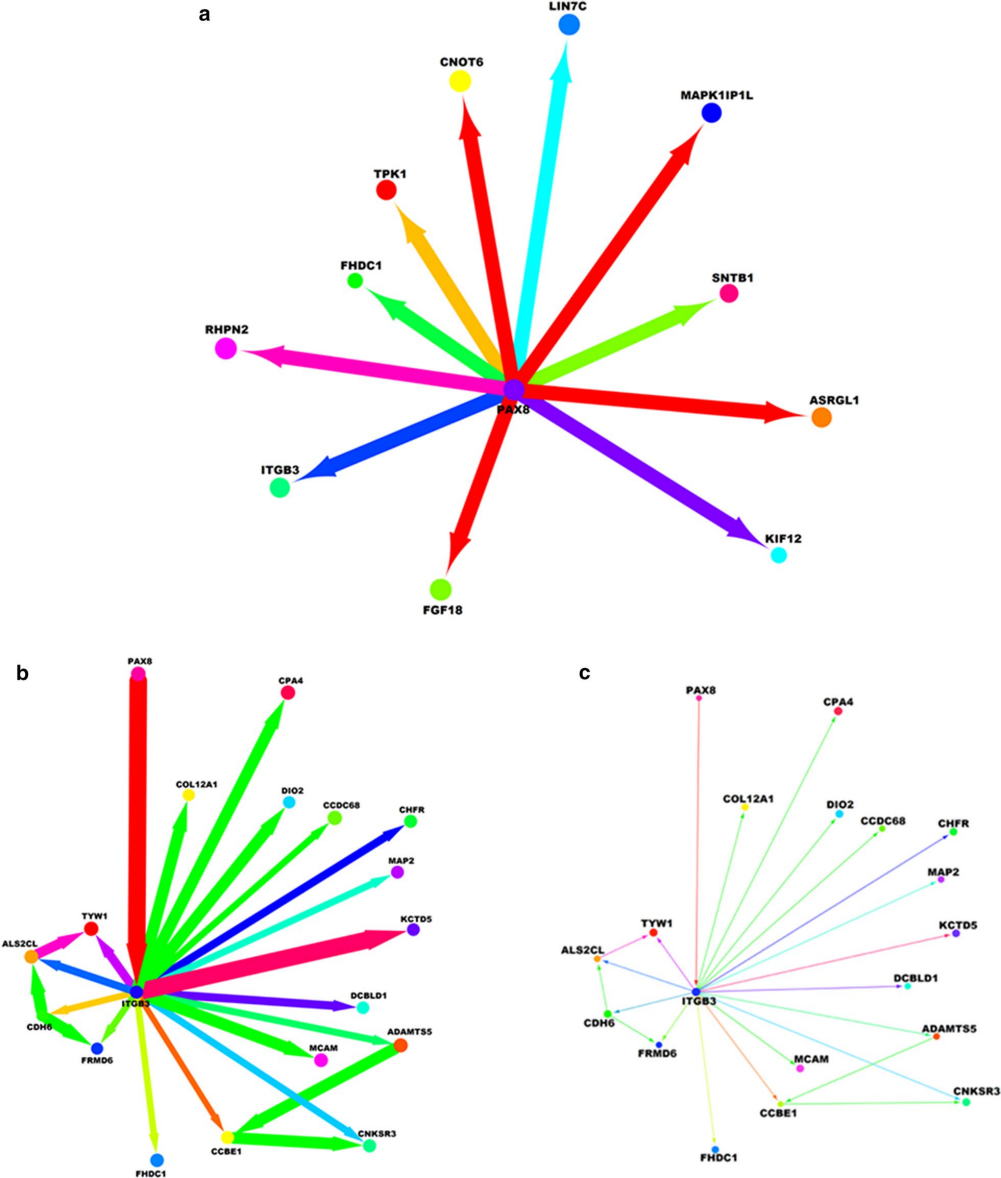
**a** Cytoscape analysis of PAX8 network in SKOV3 cells; **b** Cytoscape analysis of PAX8-ITGB3 network before PAX8 silencing in SKOV3 cells; **c** Cytoscape analysis of PAX8-ITGB3 network after PAX8 silencing in SKOV3 cells

Additional file 4: Fig. S3 represents the distribution of the molecular interactions embedded in the biological-molecular network before and after PAX8 silencing. The network generated was very large and complex showing strong gene correlation and interconnection among the molecular interactions of up-regulated and down-regulated genes. Since our goal was to understand how the PAX8-ITGB3 interaction was organized in ovarian carcinoma, we performed network analysis before and after PAX8 silencing in SKOV3, extrapolating it from the general network. We did this by examining the structure and distribution of molecular interactions of PAX8 network with its first force interactors (before and after silencing). As a validation of our experimental results, ITGB3 appeared as one of the most probable first interactors of PAX8 in SKOV3 (Fig. 6a). To have better insights into the PAX8-ITGB3 regulatory network, we performed a putative gene network analysis involving ITGB3 as a mediator of PAX8 in cell–cell interaction (contact inhibition) and cellular adhesion (tumor growth). Figure 6b shows the indirect targets of PAX8 regulated by its first interactor, ITGB3. There are two clusters of interactions, on the left and right of ITGB3 demonstrating different regulatory pathways (Fig. 6b). The targets of ITGB3 show two kinds of interactions—radially pointed outwards and circularly interlinked, both modulated parallelly by ITGB3 after its own PAX8 dependent regulation. While the small circular interaction on the left is characterized by interdependent interactions and are reported to be involved in tumor mass growth, the radially pointed groups of genes on right highlight a lack of functional interdependence. Most of these gene targets are involved in cellular invasion and tumor metastasis by various mechanisms like angiogenesis, cell migration, contact inhibition through ITGB3. It is important to note that the thickness of the arrow indicates the strength of the interaction within the network, justifying that there are biological interactions stronger than others and some biological interactions play a more important role than others.

It is further interesting to note that upon PAX8 silencing in SKOV3 all its first interactors including ITGB3 and its associated targets are muted (Fig. 6c). This is demonstrated by the decrease in cell adhesion and migration upon PAX8 silencing, which is reflected again on the network analysis of PAX8–ITGB3 where the network collapses and other interaction are formed because there is no PAX8 or ITGB3 to regulate the network.

## Discussion

The lethality of the High Grade Serous Ovarian Cancer is majorly attributed to the relapse of the cancer with > 90% succumbing after the acquisition of chemo resistance [42]. In most cases, the relapse is enhanced and the spread is highly rapid and aggressive. This is associated with features characteristic to cancer cells such as changes in adhesive properties, resistance to *anoikis* leading to EMT-MET cycling and migration to secondary sites leading to the spread of the cancer [43–45]. Although tubal origin of HGSC is popularly accepted, the retention of PAX8 from Fallopian tube until development, spread and even relapse of HGSC is not yet thoroughly explored or explained. Though several research groups have tried to unravel newer roles of PAX8 in this context [17, 20], our findings in this report associating PAX8 to functionally relevant roles in HGSC such as adhesion, migration and anoikis resistance is among the first. Further it is interesting to note that each of these features are key contributors towards high metastatic nature and therapeutic failure during the course of HGSC. Another important aspect to bear in mind is that integrin is a central factor in all these aforementioned processes such as adhesion, migration and anoikis resistance [43, 44]. As a matter of fact, these processes are intertwined in a complex signaling network with each of them involving the integrin signaling to its extra cellular matrix—functional integrin heterodimer binding to its suitable substrate for cell-adhesion and migration and aberrant integrin-ECM signaling triggers anoikis resistance and further EMT or MET depending on the cancer cell context [43, 44]. Therefore, our results showing for the first time a PAX8 dependent ITGB3 expression could positively aid in understanding why PAX8 is expressed in almost 99% HGSC patients. Indeed, this is the only study that has demonstrated a correlation between the PAX8 transcription factor and the transmembrane protein Integrin β3. Also, the direct regulation of PAX8 is not only important in controlling the protein levels of ITGB3 but also in turn affects the αvβ3 heterodimer expression on the plasma membrane which could alter the cancer cell migratory properties. In fact, our results indicate that PAX8 exhibits an effect in migration and adhesion from the secretory cells of the normal fallopian tube epithelium, development of STIC in the Fallopian tube and subsequent spread to HGSC. Our results demonstrating the preservation of PAX8 control on ITGB3 expression and the display of functional αvβ3 heterodimer on the cell surface throughout the malignancy has given critical cues for the development of HGSC and its spread. This study underlines a novel role for PAX8 beyond its current perception as an immunological marker for HGSC and the correlation PAX8-αvβ3 heterodimer highlights a possible therapeutic role for PAX8 as it appears to be an important player in enhancing the metastatic potential of HGSC cells. It is imperative to further explore this particular function of PAX8 in altering the interaction of HGSC cells with their tumor microenvironment as this transcription factor could possibly have more than one mechanism and this could be critical in improving the current therapeutic regimen.

## Conclusions

In conclusion, the evidence presented support a regulatory role for PAX8 in ovarian cancer tumor cell attachment to ECM via conferring adhesion and migration abilities and *anoikis* resistance. Further studies will pursue the identification of novel inhibitors for development of efficient blocking signals for metastasis.

## Supplementary information

**Additional file 1: Table S1.** Legend for Cytoscape analysis.

**Additional file 2: Figure S1.** (a) qRT-PCR showing the expression levels of PAX8 in the cell lines used. The values are mean ± SD of three independent experiments in duplicate, normalized by the expression of ABL and expressed as fold change with respect to hFTSEC cells, whose value was set at 1.0. (b) Western blots showing the depletion of PAX8 protein upon siRNA treatment in the cell lines used. Vinculin was used to normalize the blots.

**Additional file 3: Figure S2.** RNAi of PAX8 obtained with two independent siRNA (s15404 and s15405) that confirm the impairment of migration and adhesion.

**Additional file 4. Figure S3.** Complete biological and molecular network realized before (on the left) and after (on the right) PAX8 silencing.

## Data Availability

All data including additional information generated or analyzed during this study are included in this article. All original data are available upon request.

## Abbreviations

HGSC: High Grade Serous Ovarian Cancer
STIC: Serous Tubal Intraepithelial Carcinoma
FTE: Fallopian Tube Epithelium
Primary hFTSECs: primary human Fallopian tube secretory cells
EMT: epithelial mesenchymal transition
ECM: extracellular matrix
ITGB3: Integrin β_3_
ITGAV: Integrin α_v_
