# Supplementary material for: PAX8 expression in high-grade serous ovarian cancer positively regulates attachment to ECM via Integrin β3
Source: Cancer Cell Int. 2019 Nov 20;19:303. doi: 10.1186/s12935-019-1022-8 (PMC6865034; doi:10.1186/s12935-019-1022-8)
Supplement: Supplementary file 1 — Additional file 1: Table S1. Legend for Cytoscape analysis. [file 12935_2019_1022_MOESM1_ESM.docx]

**Additional file 1: Table S1. Legend for Cytoscape analysis**

| Node | Represent single gene/protein |
| --- | --- |
| Edge | Represent the biological and molecular interactions |
| Color node | Represent only gene family and their biological pathways |
| Size node | Represent the gene expression level (up/down regulated) or the importance and strength of a node inside specific network |
| Color edge | Represent the importance and strength of molecular interactions. The dark color represents the main biological interactions, while the light color represents the secondary biological interactions |
| Size edge/edgebetwenness | Represent the weight of interactions and the strength of molecular interactions |
| Length edge | Represent the position of node inside biological network |
| Directed network | Represent the direction of interactions between source-target |
| Degree/average_degree/degree_distribution | Represents the number of edges connected to node |
| Shortest path | Indicates the number of edges between one node to the other (also called distance) |
| Diameter | Indicates the maximum distance between two nodes of the graph |
| Level of biological analysis | Represents that there are five several levels of analysis based on the hubs interactors (directed and indirected) |
| Clustering coefficient | This parameter captures the degree to which the neighbors of a given node link to each other |
