# Supplementary figures and images for: PAX8 expression in high-grade serous ovarian cancer positively regulates attachment to ECM via Integrin β3
Source: Cancer Cell Int. 2019 Nov 20;19:303. doi: 10.1186/s12935-019-1022-8 (PMC6865034; doi:10.1186/s12935-019-1022-8)

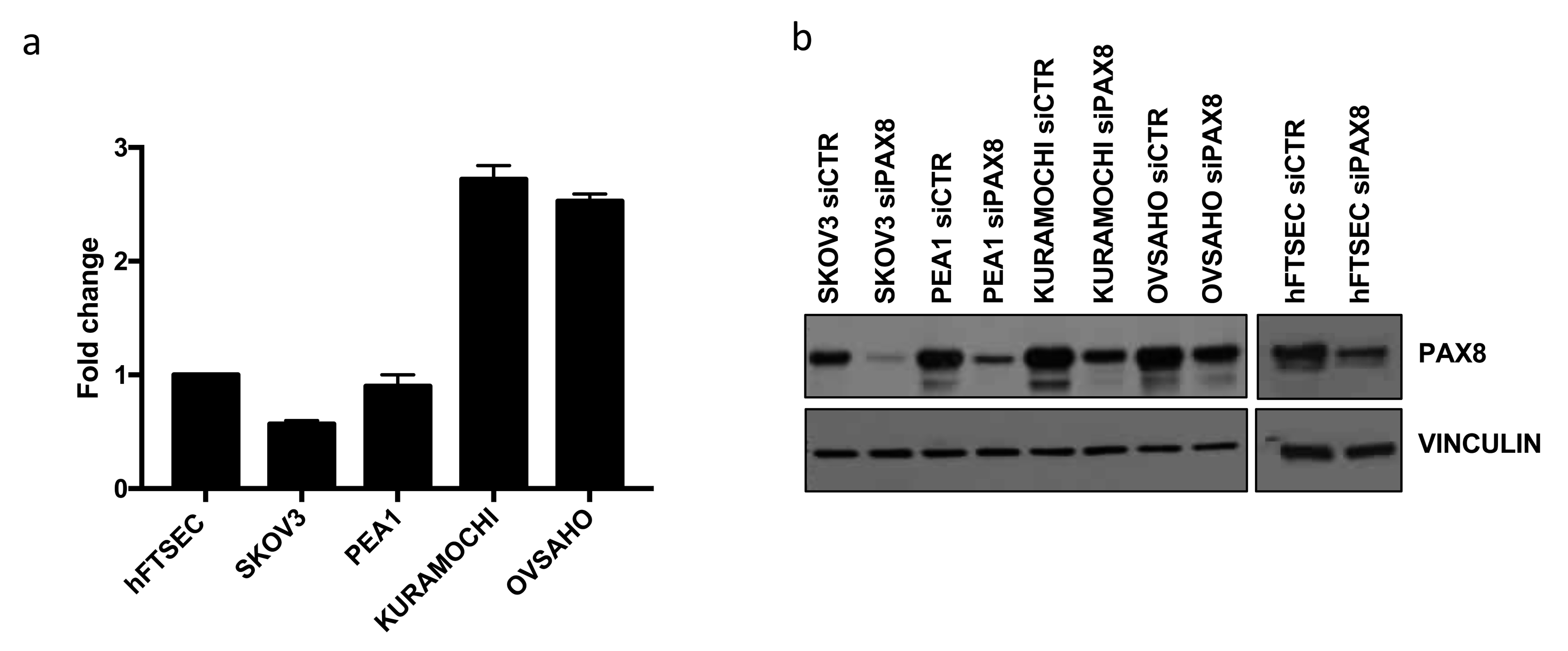

Supplement: Supplementary file 2 — Additional file 2: Figure S1. (a) qRT-PCR showing the expression levels of PAX8 in the cell lines used. The values are mean ± SD of three independent experiments in duplicate, normalized by the expression of ABL and expressed as fold change with respect to hFTSEC cells, whose value was set at 1.0. (b) Western blots showing the depletion of PAX8 protein upon siRNA treatment in the cell lines used. Vinculin was used to normalize the blots. [file 12935_2019_1022_MOESM2_ESM.tif]

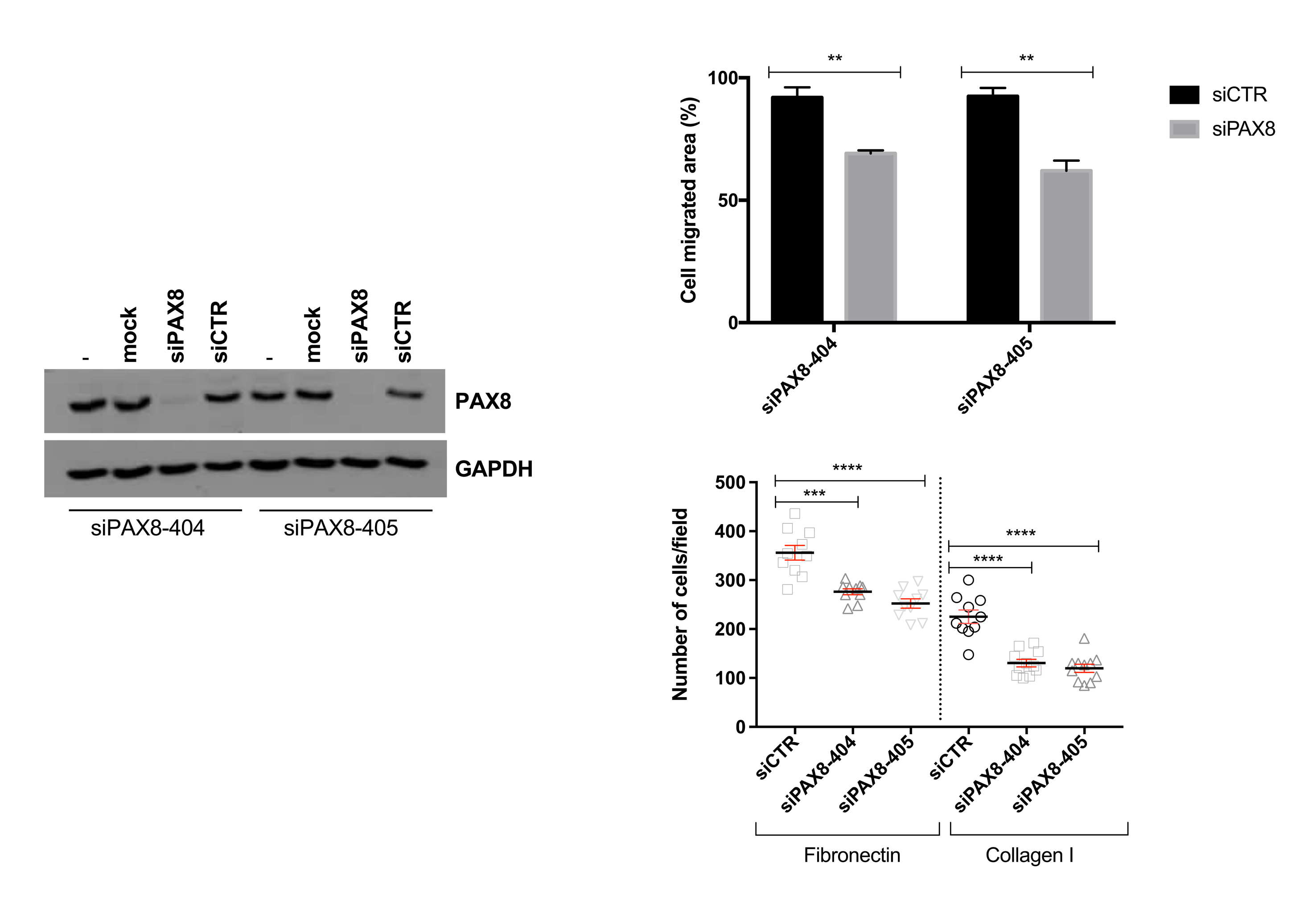

Supplement: Supplementary file 3 — Additional file 3: Figure S2. RNAi of PAX8 obtained with two independent siRNA (s15404 and s15405) that confirm the impairment of migration and adhesion. [file 12935_2019_1022_MOESM3_ESM.tif]

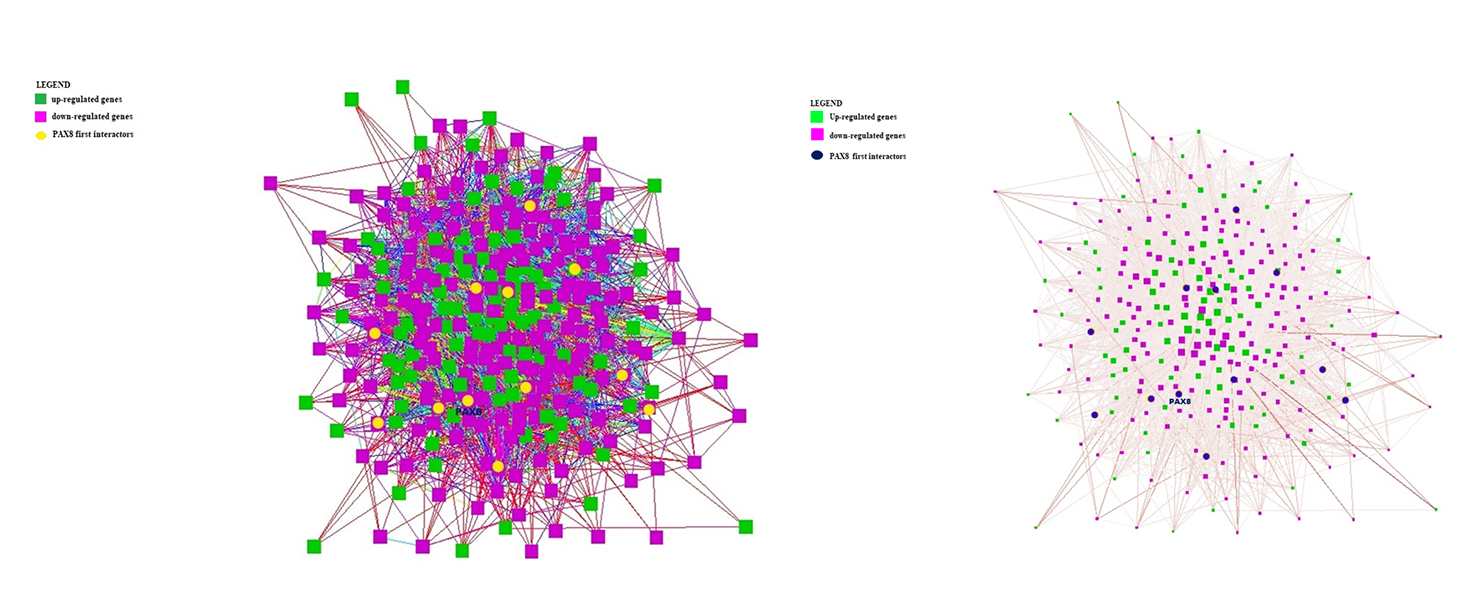

Supplement: Supplementary file 4 — Additional file 4. Figure S3. Complete biological and molecular network realized before (on the left) and after (on the right) PAX8 silencing. [file 12935_2019_1022_MOESM4_ESM.tif]
